# Complete response of mediastinal clear cell sarcoma to pembrolizumab with radiotherapy

**DOI:** 10.1186/s13569-017-0079-1

**Published:** 2017-07-14

**Authors:** Samuel Marcrom, Jennifer F. De Los Santos, Robert M. Conry

**Affiliations:** 1 Department of Radiation Oncology, University of Alabama at Birmingham, 2145 Bonner Way, Birmingham, AL 35243 USA; 2 Division of Hematology Oncology, University of Alabama at Birmingham, 2145 Bonner Way, Birmingham, AL 35243 USA

**Keywords:** Clear cell sarcoma, Radiotherapy, Immune checkpoint blockade, Pembrolizumab, Anti-PD1, Melanoma

## Abstract

**Background:**

Clear cell sarcoma (CCS) is a rare, aggressive soft tissue sarcoma thought to derive from neural crest and characterized by a 12;22 translocation. The resulting fusion protein directly activates expression of the melanocyte master transcription factor and drives the same down-stream pathways in CCS and melanoma leading to significant clinical parallels between these malignancies. Striking success of immune checkpoint blockade in melanoma has promoted interest in immunotherapy of CCS.

**Case presentation:**

We report the first complete clinical response of a bulky chest wall recurrence of mediastinal CCS in a young woman to anti-PD1 checkpoint blockade with pembrolizumab combined with standard fractionation radiotherapy to enhance regional control and potentially boost the systemic immune response. The treatment was well tolerated with grade 2 skin toxicity within the range expected with radiation alone. Significant reduction in tumor bulk occurred after only 2 radiation fractions and complete response was achieved at 50 Gray.

**Conclusion:**

The complete clinical response observed in our patient suggests synergy between concurrent radiotherapy and PD1 blockade in CCS. This case and the striking parallels between CCS and melanoma indicate the need for prospective trials of immune checkpoint blockade combined with radiotherapy in this rare malignancy.

## Background

Clear cell sarcoma, originally called “malignant melanoma of soft parts” due to its melanocytic differentiation, is a rare, aggressive soft tissue sarcoma usually arising in the lower extremities at 20–40 years of age with only three mediastinal primaries previously reported [1]. Most cases of CCS are characterized by a t(12;22)(q13;q12) translocation, resulting in fusion of the Ewing’s sarcoma gene, EWS, with the ATF1 transcription factor [2].

The EWS–ATF1 chimeric fusion protein directly activates expression of the melanocyte master transcription factor (MITF) which drives the same down-stream pathways in CCS and melanoma [3, 4]. CCS shares striking histological and immunohistochemical similarities with cutaneous melanoma, often containing melanin pigment and expressing melanocyte differentiation antigens including S-100, HMB-45, and Melan-A [5]. Like melanoma and in contrast to most sarcomas, CCS is thought to derive from neural crest, spreads to regional lymph nodes in up to 50% of patients, and is frequently associated with in transit metastases [1, 2, 5]. CCS carries a high risk of hematogeneous dissemination with 5-year overall survival of 50–60% for localized disease and poor response to chemotherapy [1, 5]. Thus, modern authors have classified CCS as a melanoma subtype [4]. Herein we report complete response of recurrent, surgically incurable CCS to the novel approach of immune checkpoint blockade with radiotherapy.

## Case presentation

A 26-year-old, pregnant, Caucasian female presented with hoarseness. Laryngoscopy demonstrated left vocal cord paralysis. After successful delivery of her baby via caesarian section, computed tomography (CT) revealed a mediastinal mass further characterized by magnetic resonance imaging (MRI) as 2.2 × 1.9 cm, abutting the left common carotid and subclavian arteries just superior to the aortopulmonary window. Positron emission tomography (PET) demonstrated a maximum standardized uptake value (SUV) of 11.8 without evidence of metastatic disease. She underwent left anterior thoracotomy to resect the mediastinal mass with negative margins.

Pathology revealed a malignant neoplasm composed of cells arranged in nests with abundant mitoses, including some with clear or vacuolated cytoplasm. Immunostains were positivity for S100, synaptophysin, SOX10, and MITF. Cytogenetics revealed a complex abnormal karyotype including t(12;22)(q13;q12) and fluorescence in situ hybridization (FISH) demonstrated an EWSR1 gene rearrangement, collectively diagnostic of CCS.

**Fig. 1:**
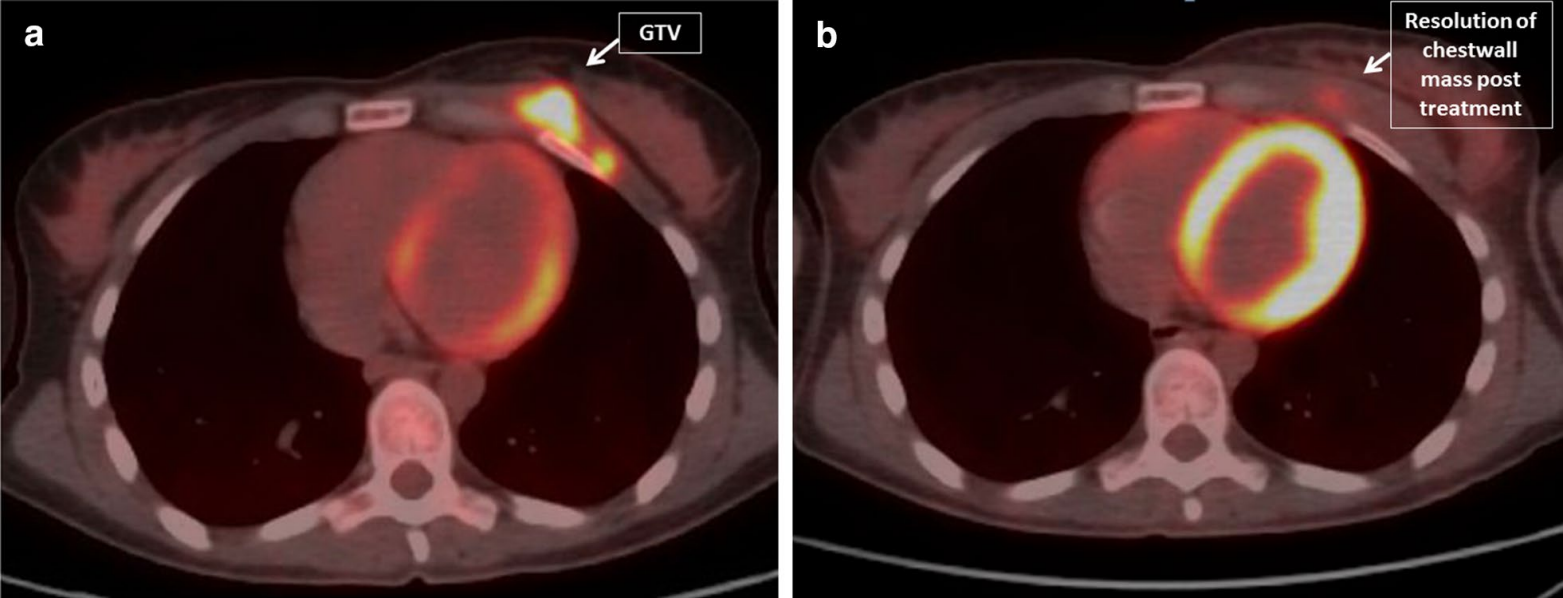
**a** PET scan prior to treatment. **b** PET scan 1 month after completion of radiation

She received adjuvant radiotherapy to the post-operative bed and regional mediastinal lymph nodes to 50 Gy in 25 fractions. Three months later, she reported rapid onset of tender enlarging masses in the left breast, and core biopsy revealed recurrent CCS. Genomic profiling with a 405 gene panel (Foundation Medicine, Inc., Cambridge, MA) demonstrated an EWSR1–ATF1 fusion characteristic of CCS; a frameshift mutation of ARID1A, a tumor suppressor gene frequently mutated in desmoplastic melanoma; and a low estimated tumor mutation burden of 2 mutations per megabase. MRI demonstrated a surgically unresectable left chest wall mass 6.7 × 2.6 cm extending into the left breast and two enhancing subcutaneous nodules just inferior to the left breast. PET/CT scan demonstrated hypermetabolic activity in these lesions with SUV max of 9.5 and no additional metastases (Fig. 1a).

Given the poor response of CCS to chemotherapy and its similarities to melanoma, immune checkpoint blockade was considered a reasonable treatment approach. Radiation therapy was proposed as an adjunctive local treatment with the goal of synergistically activating the patient’s immune system. The patient’s prior radiation, targeting the upper mediastinum, had minimal overlap with her current extent of disease. Pembrolizumab was administered at the standard dose of 2 mg/kg every 3 weeks beginning 1 week before radiotherapy. She received 50 Gy in 25 fractions to the entire left breast and involved chest wall with a 16 Gy boost in 8 fractions to the pretreatment extent of disease. Physical examination on day 10 of radiation treatment demonstrated an excellent response with a left breast index lesion decreasing from 5 to 1 cm, and a subcutaneous nodule decreasing from 1.5 to 0.5 cm. On day 18 of radiation there was no residual malignancy evident by physical exam. A re-simulation CT scan during week 5 of radiation, performed for a cone down boost to residual gross disease, revealed no evidence of residual disease. She tolerated treatment well with mild arthralgias, grade 1 esophagitis and grade 2 skin toxicity only. PET/CT 1 month after completion of radiation showed no clear evidence of malignancy, including resolution of all masses and only minimal residual hypermetabolism with SUV max of 3.0, consistent with treatment-related inflammation (Fig. 1b). At the time of this report, the patient was continuing pembrolizumab having received 6 doses without significant toxicity and remained disease-free after a complete radiographic response to the combination of pembrolizumab and radiation.

## Discussion

In this report of a young woman with recurrent, unresectable CCS, we noted a complete clinical response of bulky left chest wall disease to pembrolizumab and radiotherapy. The treatment was well tolerated with grade 2 skin toxicity within the normal range expected with radiation alone. We noted significant reduction in tumor bulk after just 2 radiation fractions, analogous to advanced melanoma treated with radiation and immune checkpoint blockade [6]. Interestingly, this was seen with standard fractionation of 2 Gy per day and resulted in a complete radiographic response at 50 Gy.

Neural crest-derived Schwann cell precursors have recently been described as bipotent stem cells which can develop into mature Schwann cells if they remain in contact with nerve fibers or melanocytes if they detach from the nerve environment [7, 8]. Malignant transformation of Schwann cells produces a malignant peripheral nerve sheath tumor, a subtype of soft tissue sarcoma, often associated with loss of function of the NF1 tumor suppressor gene. Desmoplastic melanomas with spindle cell morphology and sarcoma-like clinical behavior also frequently harbor inactivating NF1 mutations [9]. Some CCSs have electron microscopic findings suggesting Schwann cell differentiation [10]. Thus, there are multiple lines of evidence indicating embryologic and molecular genetic linkage between melanoma, clear cell sarcoma, and malignant peripheral nerve sheath tumor.

Immune checkpoint inhibitors targeting cytotoxic T lymphocyte antigen 4 (CTLA-4) or programmed death receptor 1 (PD1) have revolutionized the treatment of melanoma [11–13]. Pooled analysis from phase II and III trials of ipilimumab in 1861 patients with advanced cutaneous melanoma demonstrated 3-year survival of 26% in treatment naïve patients [11]. Single agent anti-PD1 monoclonal antibodies, nivolumab and pembrolizumab, have produced objective response rates (ORR) of 30–40% and median overall survival of 2 years in advanced cutaneous melanoma [12, 13]. Nivolumab also produced an ORR of 23% in advanced mucosal melanoma generally lacking ultraviolet-signature mutations and thus harboring fewer neopitopes [14]. Furthermore, anti-PD1 monoclonal antibodies are FDA-approved for a wide variety of malignancies including non-small cell lung cancer, renal cell carcinomas, Hodgkin’s lymphoma, and squamous cell carcinoma of the head and neck. A phase II trial of pembrolizumab in previously treated advanced soft tissue sarcoma demonstrated a partial response rate of 33% among 18 patients with undifferentiated pleomorphic sarcoma or liposarcoma with no complete responders [15].

Parallels with melanoma have promoted interest in immunotherapeutic approaches to CCS. A phase I trial of an autologous GM-CSF-secreting tumor cell vaccine in advanced CCS patients demonstrated induction of T cell-mediated delayed-type hypersensitivity reactions to irradiated, autologous tumor cells [2]. Anecdotal reports in CCS patients have included a complete response to perilesional interferon-alpha, stable disease for 6–24 months in 2 patients following ipilimumab, and a partial response to pembrolizumab in a 6-year old [5]. Single modality radiotherapy would likely have limited efficacy in bulky, unresectable CCS [16]. However, recent observations in melanoma patients have shown that anti-PD1 therapy can enhance local response to radiotherapy and promote abscopal effects with tumor regression at unirradiated sites [17]. Preclinical data and early clinical observations supporting synergy between immune checkpoint blockade and radiotherapy across multiple tumor types have been recently reviewed [18, 19].

The complete clinical response observed in our patient strongly suggests synergy between concurrent radiotherapy and PD1 blockade in CCS. This case coupled with the striking parallels between CCS and melanoma indicate the need for prospective trials of immune checkpoint blockade combined with radiotherapy in this rare malignancy striking young adults.

## Abbreviations

CCS: clear cell sarcoma
MITF: melanocyte master transcription factor
MRI: magnetic resonance imaging
PET: positron emission tomography
FISH: fluorescence in situ hybridization
CTLA-4: cytotoxic T lymphocyte antigen 4
PD1: programmed death receptor 1
ORR: objective response rates
